# The Repetitive DNA Composition in the Natural Pesticide Producer *Tanacetum cinerariifolium*: Interindividual Variation of Subtelomeric Tandem Repeats

**DOI:** 10.3389/fpls.2019.00613

**Published:** 2019-05-16

**Authors:** Jelena Mlinarec, Ana Skuhala, Adela Jurković, Nenad Malenica, Jamie McCann, Hanna Weiss-Schneeweiss, Borut Bohanec, Višnja Besendorfer

**Affiliations:** ^1^Division of Molecular Biology, Department of Biology, Faculty of Science, Zagreb, Croatia; ^2^Institute of Biotechnology, University of Natural Resources and Life Sciences, Vienna, Vienna, Austria; ^3^Department of Botany and Biodiversity Research, University of Vienna, Vienna, Austria; ^4^Biotechnical Faculty, Ljubljana, Slovenia

**Keywords:** evolution, FISH, meiosis, interstitial telomeric repeats, L-type rDNA arrangement, satellite, subtelomere, *Tanacetum cinerariifolium* (Dalmatian pyrethrum)

## Abstract

Dalmatian pyrethrum (*Tanacetum cinerariifolium* (Trevir.) Sch. Bip.), a plant species endemic to the east Adriatic coast, is used worldwide for production of the organic insecticide, pyrethrin. Most studies concerning Dalmatian pyrethrum have focused on its morphological and biochemical traits relevant for breeding. However, little is known about the chromosomal evolution and genome organization of this species. Our study aims are to identify, classify, and characterize repetitive DNA in the *T. cinerariifolium* genome using clustering analysis of a low coverage genomic dataset. Repetitive DNA represents about 71.63% of the genome. *T. cinerariifolium* exhibits linked 5S and 35S rDNA configuration (L-type). FISH reveals amplification of interstitial telomeric repeats (ITRs) in *T. cinerariifolium*. Of the three newly identified satellite DNA families, TcSAT1 and TcSAT2 are located subterminally on most of *T. cinerariifolium* chromosomes, while TcSAT3 family is located intercalary within the longer arm of two chromosome pairs. FISH reveals high levels of polymorphism of the TcSAT1 and TcSAT2 sites by comparative screening of 28 individuals. TcSAT2 is more variable than TcSAT1 regarding the number and position of FISH signals. Altogether, our data highlights the dynamic nature of DNA sequences associated with subtelomeres in *T. cinerariifolium* and suggests that subtelomeres represent one of the most dynamic and rapidly evolving regions in eukaryotic genomes.

## Introduction

Dalmatian pyrethrum (*Tanacetum cinerariifolium* (Trevir.) Sch. Bip.) is an insecticidal perennial plant of the Compositae (tribe Anthemideae). It is an outcrossing diploid (2*n* = 18) (MacDonald, 1995), self-incompatible and thermophilic plant (Parlevliet, 1975). Dalmatian pyrethrum is endemic to the east Adriatic coast with wild populations found in extremely degraded habitats with shallow rocky soils. Pyrethrum flowers produce an important insecticide, pyrethrin, which is environmentally safe and thus leading insecticide in organic farming systems (Grdiša et al., 2009). Currently, the largest producers of pyrethrum are Kenya, Tanzania, Rwanda and Tasmania (Grdiša et al., 2009). Due to the ever increasing trend of production and consummation of healthy products, coupled with an increased resistance of pests to synthetic pesticides and more strict environmental legislations, the interest in pyrethrum is higher than ever (Grdiša et al., 2009). Dalmatian pyrethrum transcriptome has been analyzed recently providing a resource for discovery of candidate genes in the biosynthesis of pyrethrin (Khan et al., 2017; Xu et al., 2018). However, little is known about the chromosome evolution and genome organization in this species.

Repetitive DNAs are the main components of the genome in higher plants. Both dispersed (DNA transposons and retrotransposons) and tandemly arranged DNA sequences (ribosomal RNA genes and satellite DNAs) are considered largely responsible for genome size variation (Garrido-Ramos, 2017). The recent advances in next generation sequencing (NGS) have revolutionized the analysis of the repetitive fraction of eukaryotic genomes (Weiss-Schneeweiss et al., 2015). RepeatExplorer is an efficient pipeline developed to allow *de novo* identification of repetitive DNA families in species lacking a reference genome (Novák et al., 2010, 2013). This approach has been efficient in the identification and characterization of repeat genome elements in several organisms (Torres et al., 2011; González et al., 2018; Yang et al., 2017).

Nuclear ribosomal RNA genes are one of the most important housekeeping genes playing a central role in cell metabolism (Grummt, 1999). Usually 5S rDNA is organized as a tandem in one to several 5S rDNA loci independent from 35S rDNA loci (S-type arrangement). Less commonly, in some lineages of seed plants, 5S rDNA monomers are in linked arrangement with monomers of 35S rDNA (L-type arrangement; Lan and Albert, 2011; Mlinarec et al., 2012; Garcia et al., 2012, 2017). L-type arrangement was first characterized in several species of the genus *Artemisia* in Asteraceae family, one of the most phylogenetically derived angiosperm groups (Yoshikazu et al., 2006; Garcia et al., 2007, 2009). Subsequent studies demonstrated that as many as 25% of Asteraceae members, including Iranian members of genus *Tanacetum*, have the L-type arrangement of rDNA (Garcia et al., 2010; Mazzella et al., 2010; Olanj et al., 2015).

Satellite DNA (satDNA) families are the main component of the heterochromatin, often localized in pericentromeric and subtelomeric regions of the chromosomes, but also found at interstitial locations of chromosomes (Mlinarec et al., 2009). Initially considered to be “junk DNA,” previous studies show that satDNAs might perform important functions in regulation of gene expressions, or play important structural role in vital functions including, among others, chromosome segregation and preservation of genetic material (Blackburn, 2005; Louis and Vershinin, 2005; Riethman et al., 2005; Kuo et al., 2006). Although subtelomeric repeat sequences have been reported in numerous plant species, their structure and organization has been analyzed in detail in only a few species (Vershinin et al., 1995; Cheng et al., 2001; Dechyeva and Schmidt, 2006; Torres et al., 2011; Richard et al., 2013; Chen et al., 2018). The mechanisms by which these repeats are maintained and evolve are not well understood.

Telomeres in most eukaryotes consist of short, tandemly repeated DNA sequences. In plants, *Arabidopsis*-type (TTTAGGG)_n_ is the dominant consensus telomere repeat (Richards and Ausubel, 1988). ITRs are localized within interstitial regions (ITRs) on chromosomes. Their origin largely remains unknown, however, it was suggested that ITRs might originate from ancestral intrachromosomal rearrangements involving telomeric regions (inversions and fusions), unequal crossing-over or from the repair of DSBs. ITRs have been reported in various organisms, including vertebrates (Meyne et al., 1990) and plants (Cox et al., 1993; Fuchs et al., 1995; Rosato et al., 2018).

The goals of our study were to: (a) identify and classify repetitive elements in the *T. cinerariifolium* genome by the cluster analysis using RepeatExplorer, to (b) analyze the chromosomal distribution patterns of major tandem repetitive DNA families and to (c) examine the patterns of variation at the chromosomal subtelomeric regions in natural and cultivated *T. cinerariifolium* populations by comparative screening of 28 individuals.

## Materials and Methods

### Plant Material

*Tanacetum cinerariifolium* (Trevir.) Sch. Bip. (2*n* = 2*x* = 18) (accession 53-3) was used for Illumina NextSeq genome sequencing (“genome skimming”). Voucher for that accession was preserved as herbarium specimen at Herbarium Croaticum of the University of Zagreb (ZA) under the number ZA47972. Thirty four accessions belonging to five populations were used for cytogenetic studies. Table 1 lists the provenance of the studied materials. Seeds were collected in the field from four natural populations from Croatia: Konavle (425506N, 0172352E), Osor (445542N, 0145519E), Cres-Merag (443150N, 0142807E), Cres-Kimen (445739N, 142428E). Seeds from Tanzania, originating from commercial crop were purchased from the international trade association CropLife International A.I.S.B.L. (Brussels, Belgium) by the courtesy of Dr. David Wantenaar. After germination, seedlings were grown individually in a mix of Substrat2 potting substrate (Klasmann-Deilmann GmbH Germany) and siliceous sand (3:1) in the greenhouse of the Faculty of Science of University of Zagreb under 16 h of light and 8 h of the dark period at 24°C until plants were large enough to be repotted.

**Table 1:** Accession number, location, geographic origin and number of rDNA and satellite DNA sites in plants investigated in this study.

Accession number	Location/population	35S/5S rDNA	TcSAT1	TcSAT2	TcSAT3	1C/pg
2143-1	Osor		28+2^∗^	22	4	
2143-2	Osor		28+1^∗^	21		9.42
2143-3	Osor	6	27+1^∗^	21	4	9.57
2143-4	Osor		27+2^∗^	21		9.54
2143-5	Osor		27+1^∗^	20		9.52
1-3	Konavle	5				
1-4	Konavle				4	
20-7	Konavle	5			4	
53-3	Konavle	6	26	14		9.73
32-2	Konavle		27	12	4	9.54
2146-1	Konavle		25	11		
2146-2	Konavle		26	10		
2146-3	Konavle		26	14		
2146-4	Konavle		26	13		9.77
2146-5	Konavle		26	14		
2146-6	Konavle	6			4	
2157-1	Cres-Merag		29	15		9.38
2157-2	Cres-Merag		26	19		
2157-3	Cres-Merag		27	18		9.36
2157-4	Cres-Merag		26	21		
2157-5	Cres-Merag		27	15		9.68
2157-6	Cres-Merag		28	18	4	
2158-1	Cres-Kimen		28	18		
2158-2	Cres-Kimen		27	17		9.50
2158-3	Cres-Kimen		28	19		
2158-4	Cres-Kimen	5			4	
2158-5	Cres-Kimen	6			4	
A-1	Tanzania		30	12		9.78
A-3	Tanzania		27	14		9.69
A-5	Tanzania		27	18		
A-6	Tanzania		28	20		
A-7	Tanzania		26	16		
A-9	Tanzania		28	16		
A-12	Tanzania		28	14		

^∗^*interstitial TcSAT1 signals*					Average	9.58
					STDEV	0.14

### Next Generation Sequencing, Pre-processing Data, and Clustering Analysis

The total genomic DNA of *T. cinerariifolium* used for NGS was isolated using DNA-GenElute Plant Genomic DNA Miniprep Kit (Sigma Aldrich, Steinheim, Germany), according to manufacturer’s instructions. NGS library was prepared using TruSeq DNA PCR-Free LT Library Prep Kit (Illumina^®^, United States). Sequencing was performed on a NextSeq 500 System (Illumina^®^, United States) at the Bart’s and the London Genome Centre, using 150 bp paired-end reads. After the quality filtering (quality cut-off value: 20; percent of bases in sequence that must have quality equal to/higher than the cut-off value: 90) the reads were subjected to similarity-based clustering analysis using RepeatExplorer (Novák et al., 2010, 2013). Reads within clusters were assembled into contigs. Clusters containing at least 0.01% of all clustered reads were manually annotated based on the similarity search results from RepeatMasker^1^ against Viridiplantae database and blastn and blastx (Altschul et al., 1990) against GenBank nr (Benson et al., 2009), which are part of the RepeatExplorer output. The classification integrated in the RepeatExplorer was used (Neumann et al., 2019). Clusters of annotated putative mitochondrial and plastid contaminations were filtered out. Clusters containing satellite repeats were identified based on graph structure and using DOTTER (Sonnhammer and Durbin, 1995). Reconstruction of monomer sequences of individual satellite DNA families was performed using TAREAN (Novák et al., 2017), integrated in RepeatExplorer pipeline. Search for distinctive motifs typical of retroelements in 5S rDNA was conducted using Blast at “DPTEdb: Dioecious Plants Transposable Elements Database^2^.” Genome coverage was calculated as follows: coverage = (r × l)/g, where r corresponds to number of reads used in our analysis, l to read length and g to haploid genome size of *T. cinerariifolium*.

### DNA Extraction, Primer Design and PCR Amplification of TcSAT1, TcSAT2, and TcSAT3 Satellite DNA Families

Genomic DNA was isolated from young leaves (population Konavle, accession 53-3) using the Qiagen mini-kit (Hilden, Germany) according to the manufacturer’s instructions. Satellite monomers TcSAT1, TcSAT2, and TcSAT3 used as probes for FISH, were amplified with specific primers designed based on contig recovered from clustering analyses using program Primer3 (v. 0.4.0) (Koressaar and Remm, 2007; Untergasser et al., 2012) (Table 2). The satellite DNA monomers were amplified by PCR. All PCRs were performed using Q5^®^ High-Fidelity PCR kit (New England Biolabs, Inc., United States): 1X Q5 High-Fidelity Master Mix, 10 pmol of each primer (Macrogen, Amsterdam, The Netherlands) and 1 μl of template DNA (16 ng), in a 25 μl final reaction volume. PCR program consisted of 35 cycles, each with 1 min denaturation at 95°C, 10 s annealing at 58°C, 1 min extension at 72°C, and a final extension of 7 min. Amplification products were separated by electrophoresis on a 1% agarose gel, stained with Syber Safe (Invitrogen, Eugene, OR, United States), and visualized with UV transilluminator.

**Table 2:** Primer sequences for amplification of tandem repeat specific probes.

Primer	Repeat family	Sequence (5′-3′)
TcSAT1_F	TcSAT1	CGTTGATTTCTCTTTGCTTGG
TcSAT1_R	TcSAT1	TTGGGATCGTTTAGGGCTTT
TcSAT2_F	TcSAT2	CAACCCTCAAATGTGAAAACC
TcSAT2_R	TcSAT2	CTGACAATCGGGATCTTTCC
TcSAT3_F	TcSAT3	GGGTCATTGGTTTGAGTTGG
TcSAT3_R	TcSAT3	TTGATCACAGTGCATAATTTGG

*F, forward primer; R, reverse primer.*

The sequences of the amplified monomers were verified by cloning of the PCR product into pGEM-T Easy vector according to the manufacturer’s instruction (Promega, Madison, WI, United States). The individual clones were sequenced by Macrogen (Amsterdam, Netherlands).

### Preparation of Chromosome Spreads

Mitotic metaphase chromosomes used for FISH assay were prepared following the published protocols (Mlinarec et al., 2006). Actively growing root-tip meristems were pre-treated with an aqueous solution of 0.05% colchicine (Sigma-Aldrich Chemie GmbH, Taufkirchen, Germany) in darkness for 4 h at room temperature and fixed in 3:1 (v/v) ethanol: acetic acid. Flower buds were directly fixed in the same fixative solution. To avoid mechanical pressure (squash) in preparation of chromosome spreads, the pollen mother cells were isolated by gentle squeezing of anthers with needle in 45% acetic acid. After removal of anther tissue, the material was covered with coverslip and briefly warmed over the flame. The slides with meiocytes in the stages from pachytene to diakinesis were selected by inspection under the phase contrast microscope prior to removal of the coverslip by ice-dry. The slides were dried overnight at room temperature (RT).

### Fluorescence *in situ* Hybridization (FISH)

The purified 472 bp insert (GenBank JX101916.1) from the plasmid carrying a 5S rRNA gene trimer from *Artemisia tridentata* (clone 4T) was used as the 5S rDNA probe (Garcia et al., 2012). The 2.4 kb *Hind*III fragment of the partial 18S rDNA and ITS1 from *Cucurbita pepo*, cloned into pUC19 vector (Torres-Ruiz and Hemleben, 1994), was used as the 35S rDNA probe. The *Arabidopsis*-type telomeric DNA was generated by PCR amplification in the absence of template using primers (TTTAGGG)_4_ and (CCCTAAA)_4_ according to Ijdo et al. (1991). The probes used to map satDNAs in the chromosomes were DNA fragments obtained by PCR. Total PCR products were purified using Wizard^®^ SV Gel and PCR Clean-up System (Promega Corporation, Madison, United States) and directly labeled with either Aminoallyl-dUTP-Cy3 (Jena Bioscience GmbH, Jena, Germany) or with Green-dUTP (Abbott Molecular Inc., United States) using Nick Translation Reagent Kit according to the manufacturer’s instructions (Abbott Molecular Inc., United States) with some modifications. 500 ng of probe was labeled in a total volume of reaction of 25 μl using 2.5 μl of enzyme mixture for 6 h at 15°C. FISH was performed according to Mlinarec et al. (2012) with some modifications. The slides were fixed for 10 min in 3.7% formaldehyde in 2 × SSC (standard saline citrate) with 0.1% (w/v) sodium dodecyl sulfate. The hybridization mixture (20 μl) containing 50% formamide, 10% dextran sulfate, 0.6% sodium dodecyl sulfate, 2 × SSC and 2 ng/μl of labeled probe was denatured at 96°C for 3 min and immediately cooled on ice. Chromosome preparations were denatured together with hybridization mixture at 73°C for 5 min. Stringent washes were performed at 42°C in the following solutions: 2 × SSC, 0.1 × SSC, 2 × SSC, 4 × SSC/Tween, 5 min each. Slides were counterstained with DAPI (2 μg/ml) for 20 min at RT.

The preparations were mounted in Dako Fluorescence Mounting Medium (Dako North America Inc., United States) and stored at 4°C overnight. Signals were visualized and photographs captured using an Olympus BX51 microscope, equipped with a cooled CCD camera (Olympus DP70). Single channel images were overlaid and contrasted using Adobe Photoshop 6.0 with only those functions that apply to the whole image. An average of 10 well-spread metaphases was analyzed for each individual.

### Flow Cytometry Analysis

For determination of relative nuclear DNA content four leaf samples were analyzed per each accession as described earlier (Bohanec, 2003). Nuclei of leaf samples together with internal standard *Pisum sativum* cv. Kleine Rheinländerin (9.07 pg/nucleus) were released in 0.1 M citric acid containing 0.5% Tween 20. The suspensions were filtered through 30 μm nylon-mesh filters. 3–4 vol of staining buffer containing 4 μg ml-1 4′,6′-diamidino-2-phenylindole (DAPI) in 0.4 M disodium hydrogen phosphate were added to each sample. Measurements were done on Partec CyFlow^®^ Space flow cytometer using linear scale. Relative nuclear DNA content was calculated using FloMax^®^ software (Partec, Münster, Germany).

### Accession Codes

Cloned sequences of satellite repeats were deposited in genBank under accession numbers MK330222 for TcSAT1, MH663528 for TcSAT2 and MH663529 for TCSAT3. Raw Illumina reads from DNA-seq experiment is available from European Nucleotide Archive under the study PRJNA524009.

## Results

### *Tanacetum cinerariifolium* Repeatome Characterization and Identification of Tandem Repeats

The genome size of *T. cinerariifolium* was measured in 13 individuals from five populations. Results showed that the average 1C DNA value was 9.58 ± 0.14 pg (Table 1). To evaluate the global repeat composition of the *T. cinerariifolium* genome, a combined dataset of 2 064 920 randomly selected reads (coverage of 0.033×) was used as input in the cluster analysis using the RepeatExplorer pipeline. Clustering of 1 706 405 paired-end reads resulted in 58 204 clusters. The nuclear repetitive DNA constituted 71.63% of the genome (Table 3). Retrotransposons were the most abundant elements, representing 54.23% of the genome, with *gypsy* elements contributing 30.3% (*Athila, Tekay, TatV*, and *Reina* lineages), and *copia* elements contributing 23.94% (*SIRE, Angela, TAR, Ale*, Tork, and *Ivana*). DNA transposons constituted very low portion of the genome (0.29%), while rRNA genes (5S and 35S rDNAs) represented 0.25% of the genome (Table 3).

**Table 3:** Major types of repetitive DNA in *T. cinerariifolium*.

Subgenus/Section	%
Satellites	
*TcSAT1*	0.88
*TcSAT2*	0.12
*TcSAT3*	0.02
Total satellites	1.04
LTR elements	
*Ty3/gypsy*	
*Athila*	16.33
*Chromovirus*	13.66
*TatV*	0.3
Total Ty3/gypsy	30.3
*Ty1/copa*	
*SIRE*	22.15
*Angela*	1.03
*TAR*	0.23
*Ale*	0.23
*Tork*	0.16
*Ivana*	0.13
Total Ty1/copia	23.94
Other repetitive elements	
LINE	0.25
DNA transposon	0.29
rDNA	0.25
Pararetrovirus	0.21
Unclassified repetitive	15.36
Low and single copy	17.68
Total % repetitive DNA	71.63

**FIGURE 1:**
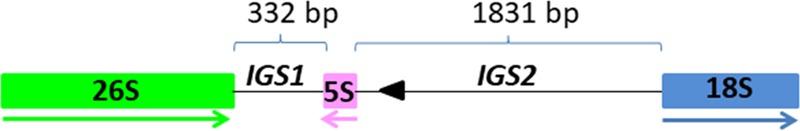
The position and organization of 5S insertion within the 26S-18S intergenic spacer. The 26S, 5S, and 18S genic regions are in green, pink and blue boxes, respectively. The size of IGS1 and IGS2 are depicted with thin lines above the units. Direction of transcription is illustrated by thick arrows below the genes. Arrowhead indicates sequence homologous to MITE family DTM_Glm17 from *Glycine max*.

Three clusters - CL14, CL82 and CL153 - were identified as tandemly repeated satellite DNAs based on the shape of the output graphs and have been designated as TcSAT1, TcSAT2, and TcSAT3 (stands for TcSAT1 through to 3 in decreasing genomic abundance (Supplementary Figure S1). Their consensus monomers sequences were reconstructed (Supplementary Figures S2, S3). TcSAT1 represented 0.882% of the genome, with consensus monomer sequence of 273 bp in length and 33.7% of GC content (Table 4). TcSAT2 represented 0.116% of the genome with consensus monomer sequence of 897 bp and 29.43% of GC content (Table 4). TcSAT3 represented 0.016% of the genome with consensus monomer sequence of 1120 bp and 29.59% of GC content (Table 4). TcSAT1 sequences did not show similarity to the TcSAT2 and TcSAT3 repeats. The GenBank and PlantSat (Macas et al., 2002) databases-based BLAST analysis showed no homology with any other known repeats. TcSAT1 and TcSAT3 had no identifiable subrepeats while some subrepeats (albeit not clearly structured) were found in TcSAT2 (Supplementary Figure S4). Only one monomer of the perfect telomeric sequence motif (T_3_AG_3_) was present within each TcSAT1 and TcSAT3 monomers, while TcSAT2 monomer contained no telomeric sequence motifs. Imperfect telomere-like sequences were more abundant in all three of the satellite DNAs. In TcSAT1 and TcSAT3 these motifs were largely dispersed over the whole monomer length), while in TcSAT2 a larger cluster of cca. 21 bp has been found. The cluster CL48 contained both 5S rDNA and 35S rDNA with 5S rDNA inserted within the IGS region (Figure 1). 5S rDNA was inserted within IGS in inverted transcription orientation with respect to that of the 35S rDNA. According to the position of 5S rRNA IGS is divided into IGS1 and IGS2. IGS1 was 332 bp long, and IGS2 was 1831 bp long. Sequences flanking 5S rDNA insertions were searched for distinctive motifs typical of retroelements such as short repeats, long terminal repeats (LTR) or transcription priming sites. IGS of *T. cinerariifolium* contained 34 bp long motif resembling to MITE family DTM_GLM17 from *Glycine max*. The motif was positioned 166 bp downstream from the 5S rDNA genic region. Duplications of a putative integration site as well as domains typical from autonomous retrotransposons (e.g., reverse transcriptase) were not found.

**Table 4:** Characterization of satellite DNA families TcSAT1, TcSAT2, and TcSAT3 in *T. cinerariifolium* genome.

Repeat family	Monomer length (bp)	GC-richness (%)	Satellite probability^∗^	% Genome^∗∗^
TcSAT1	273	33.7	0.994	0.882
TcSAT2	897	29.43	0.986	0.116
TcSAT3	1120	29.59	0.994	0.016

*^∗^As estimated by TAREAN (tool for identification of satellite DNAs in RepeatExplorer pipeline). ^∗∗^given as the output of RepeatExplorer analyses.*

### Chromosomal Distribution of Tandem Repeats Revealed by FISH

**FIGURE 2:**
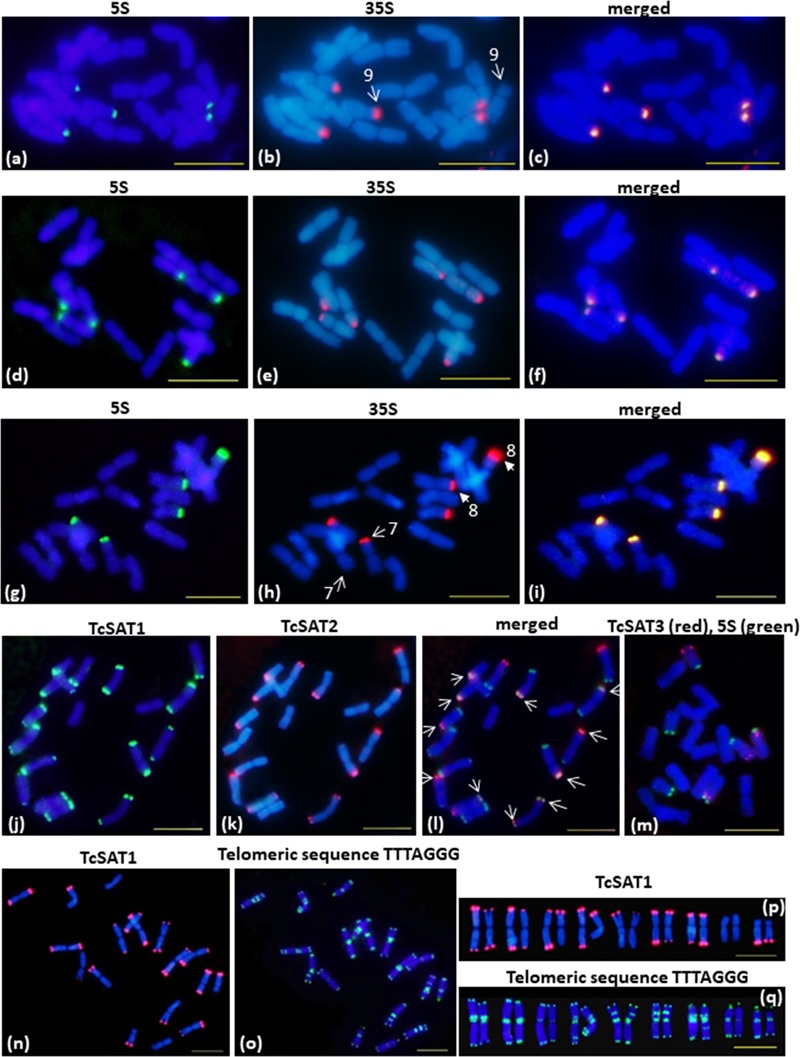
FISH mapping of 35S/5S rDNA, TcSAT1, TcSAT2 and TcSAT3 satellite DNA repeats and *Arabidopsis*-type (TTTAGGG)_n_ telomeric repeats on somatic metaphase chromosomes of *Tanacetum cinerariifolium* (2*n* = 18). **(a–i)** FISH on metaphase chromosomes that evolved linked arrangement of rRNA genes. **(a–c)** accession 20-7 and **(g–i)** accession 2158-4 have five linked 5S/35S rDNA sites. **(d–f)** accession 53-3 has six linked 5S/35S rDNA sites. Arrows on **(b,h)** mark heteromorphic chromosome pairs 9 and 7 and 8, respectively. The 35S and 5S rDNA loci are labeled in green and red, respectively. **(j–l)** FISH mapping of TcSAT1 (green) and TcSAT2 repeats (red). Overlapped TcSAT1 and TcSAT2 signals are marked with arrows. **(m)** FISH mapping of TcSAT3 repeat family (red) and 5S rDNA (green). **(n–q)** FISH mapping of the TcSAT1 (red) and *Arabidopsis*-type plant telomeric DNA probe (green). **(j–l), (n–q)** accession 53-3. **(m)** accession 2158-5. Scale bar, 10 μm.

Chromosomal distribution of rDNA loci have been investigated in seven individuals belonging to three populations (Osor, Konavle and Cres-Kimen) (Table 1). The FISH assay confirmed L-type organization of ribosomal RNA genes of *T. cinerariifolium* (2*n* = 2*x* = 18) revealed from cluster analysis by RepeatExplorer. Three 35S/5S rDNA (35S/5S) loci were located subterminally on short arms of the chromosomes 7, 8, and 9 (Figure 2a–i). All three 35S/5S loci were polymorphic. Chromosome 8 was polymorphic in regard to the size and intensity of FISH signal, while chromosomes 7 and 9 were polymorphic in regard to the presence/absence of FISH signal between seven individuals analyzed (Figure 2b,h, arrows).

**FIGURE 3:**
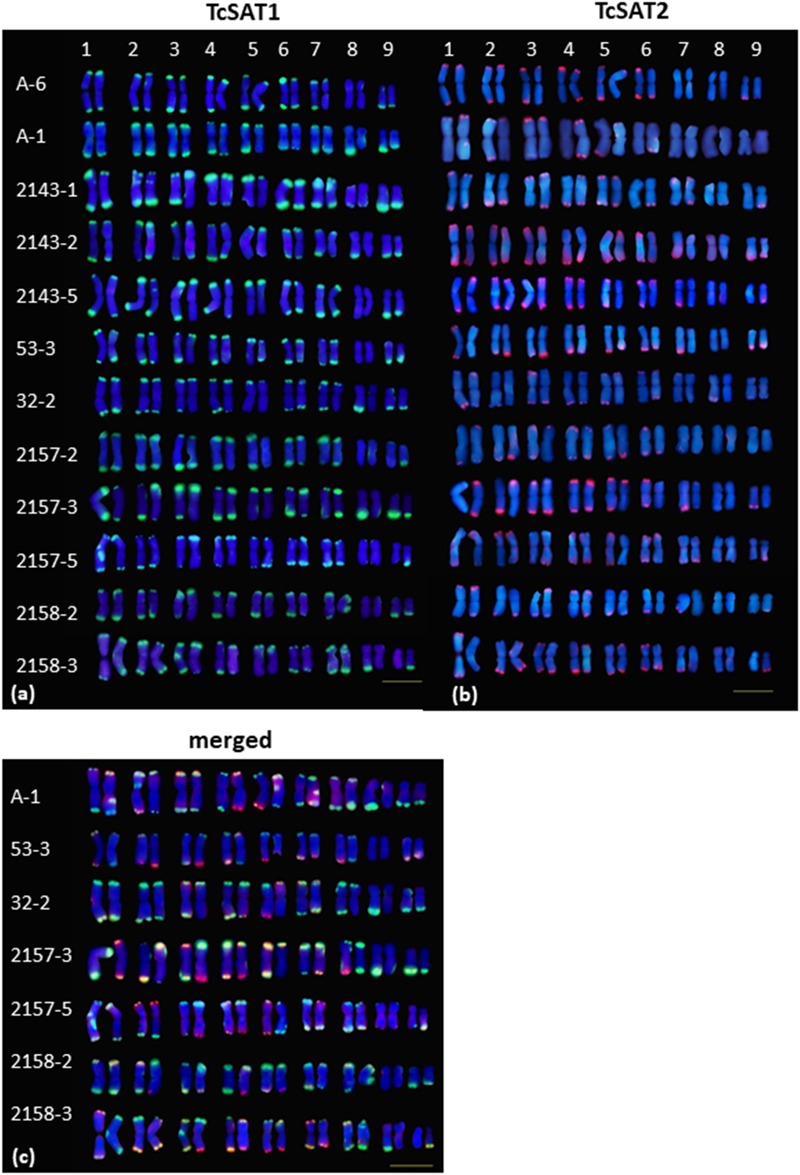
Polymorphism of the TcSAT1 (green) and TcSAT2 (red) sites revealed by FISH. Homologous chromosome pairs were identified based on the morphology and FISH signal patterns and arranged according to their sizes. Accession number is left to the kariograms. Scale bar, 10 μm.

Chromosomal localization of TcSAT1 and TcSAT2 satellite repeats was investigated in 28 individuals from five populations, while TcSAT3 satellite repeat family chromosomal distribution has been investigated in nine individuals belonging to four populations (Osor, Konavle, Cres-Merag and Cres-Kimen) (Table 1). FISH analysis revealed that two AT-rich satellite DNA families, TcSAT1 and TcSAT2, were located almost exclusively at the distal ends of majority of *T. cinerariifolium* mitotic metaphase chromosomes (Figure 2j–l), while the satellite TcSAT3 was localized in intercalary positions of two NOR-bearing acrocentric chromosome pairs 7 and 8 (Figure 2m). The only exception was population Osor in which interstitial TcSAT1 signal was observed on the short arm on either one or both chromosomes of the pair 2 (Table 1 and Figure 3a). The subtelomeric satellite DNA families TcSAT1 and TcSAT2 were heterochromatic as confirmed by counterstaining with DAPI after FISH (Supplementary Figure S5). The amount of the TcSAT1 and TcSAT2 at different chromosomal ends varied as inferred from the sizes and intensities of the FISH signals (Figure 2p, 3). FISH revealed no polymorphism of TcSAT3 signals among the nine individuals investigated in this study (Table 1). When the TcSAT1 and TcSAT2 repeats were co-hybridized on somatic metaphase chromosomes, both repeats hybridized subterminally to the majority of chromosomes (Figure 2l, arrows). The subtelomeric locations of TcSAT1 and TcSAT2 satellites were confirmed by co-hybridization with the *Arabidopsis*-type telomeric DNA probe (Figure 2n–q).

Telomeric repeat distribution has been investigated in 11 individuals belonging to three populations (Konavle, Cres-Merag and Tanzania). FISH experiments using the *Arabidopsis*-type telomeric probe (TTTAGGG)_n_ revealed strong and consistent signals in the terminal ends of both chromosomal arms of all *T. cinerariifolium* chromosomes (Figure 2o,q). In addition, telomeric probe hybridized also to pericentromeric and/or ITRs of nearly all chromosomes. ITR signals were of different sizes and intensity and the majority of ITR signals were more intense than the signals in the terminal chromosome ends. Every chromosome pair within the complement exhibited unique ITRs pattern. Pericentromeric ITRs were observed on all chromosomes, while interstitial ITRs were observed on five chromosome pairs (1, 4, 5, 6 and 7). FISH revealed no polymorphism regarding the presence/absence of telomeric signals among 11 individuals investigated.

### Polymorphism of the TcSAT1 and TcSAT2 Sites

The localization of TcSAT1 and TcSAT2 was analyzed in 28 individuals belonging to five populations. FISH revealed high levels of polymorphism of the TcSAT1 and TcSAT2 sites (Table 1 and Figure 3). TcSAT2 was more variable than TcSAT1 regarding the number and position of FISH signals (Table 1 and Figure 3). The number of TcSAT1 sites ranged from 26 to 30, while number of TcSAT2 sites ranged from 10 to 22 (Table 1). The highest variation of the numbers of TcSAT1 and TcSAT2 signals was present in the cultivated plants from Tanzania in which 26 to 30 TcSAT1 and 12 to 20 TcSAT2 signals were observed. The Osor population was the least variable with 28 to 29 TcSAT1 and 20 to 22 TcSAT2 signals observed. All five investigated individuals from Osor population possessed unique interstitial TcSAT1 signal on chromosome pair 2 not observed in any other investigated individual from other populations (Figure 3a).

**FIGURE 4:**
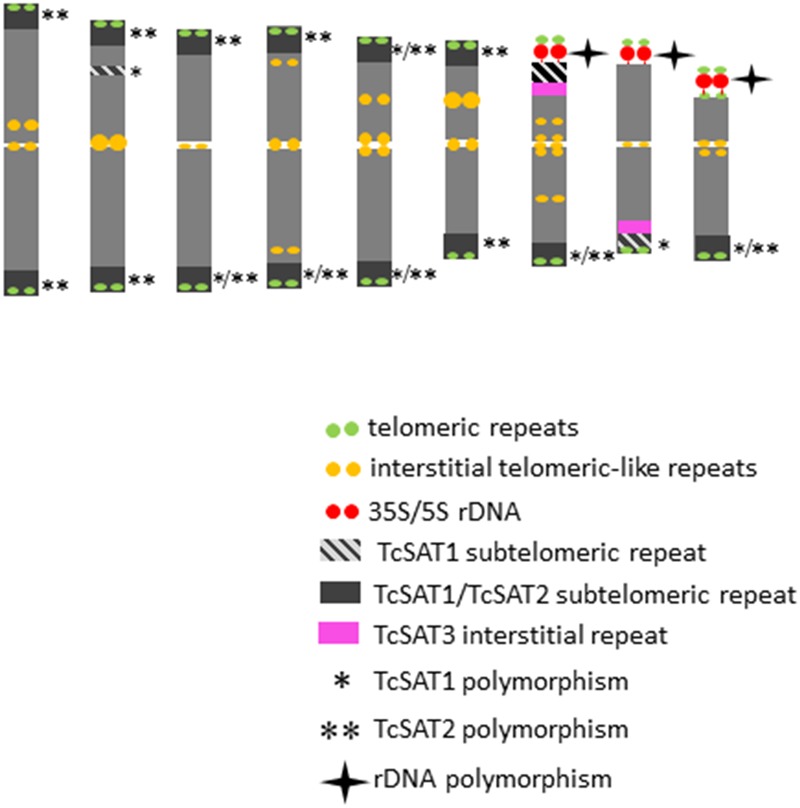
An ideogram of *T. cinerariifolium* chromosomes with marked localization of 35S/5S rDNA, *Arabidopsis*-type (TTTAGGG)_n_ telomeric repeats, as well as TcSAT1, TcSAT2 and TcSAT3 satellite repeats. Polymorphic TcSAT1 and TcSAT2 sites are marked by asterisk and double asterisk, respectively. Polymorphic rDNA sites are marked with star. This ideogram was generated based on the FISH information from Figure 2.

The detailed analyses of the chromosomal distribution of polymorphic satDNAs sites were performed in 12 individuals belonging to five populations (Figure 3). Chromosomes with FISH signals were arranged into karyograms according to their size and morphology and guided by the telomeric probe hybridization patterns. Cytogenetic analysis revealed high levels of variation in the position of TcSAT1 and TcSAT2 signals with each analyzed individual possessing unique TcSAT1 and TcSAT2 chromosomal pattern. Furthermore, every individual was heterozygous for at least one of TcSAT1 and TcSAT2 loci. Overall, eight polymorphic TcSAT1 loci and 14 polymorphic TcSAT2 loci have been found in the 12 individuals (Figure 3, 4). Polymorphic TcSAT1 loci were located subterminally on long arm of chromosomes 3, 4, 7, 8 and 9, on both chromosomal arms of chromosome 5, and interstitially on short arm of chromosome 3. Polymorphic TcSAT2 loci were located on both arms of chromosomes 1, 2, 3, 4, 5 and 6, and on long arms of chromosomes 7 and 9. In general, TcSAT1 tended to be more often present at the ends of the shorter arms, whereas TcSAT2 was equally present at the ends of the both chromosome arms (Figure 3, 4).

When the two satellite repeats, TcSAT1 and TcSAT2, were co-hybridized to the same mitotic metaphase cells of seven individuals, they produced a total from 28 to 31 subtelomeric signals (Figure 3c). 11 to 14 chromosomes had signals in both arms, while two to six chromosomes had signal on one chromosomal arm. Only five of the 36 chromosomal ends lacked unambiguous TcSAT1 or TcSAT2 signals in somatic metaphase chromosomes of all individuals analyzed. The subtelomeres devoid of TcSAT1/TcSAT2 signals were at the end of the short arms of the chromosomes 8 and 9, and at the long arm of one chromosome of the pair 8. The localization of 35S/5S rDNAs and *Arabidopsis*-type (TTTAGGG)_n_ telomeric repeats, as well as the localization of subtelomeric TcSAT1 and TcSAT2 and interstitial TcSAT3 on *T. cinerariifolium* chromosomes are summarized in Figure 4.

**FIGURE 5:**
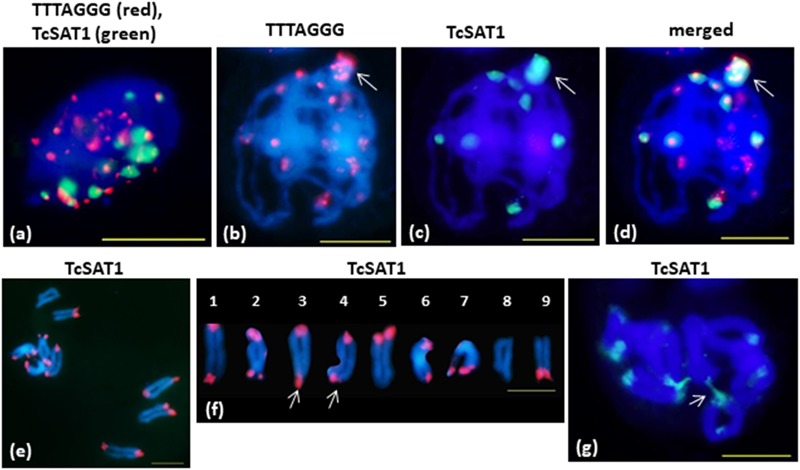
FISH mapping of TcSAT1 and TcSAT2 satellite DNA repeats and *Arabidopsis*-type (TTTAGGG)_n_ telomeric repeats on meiotic pachytene **(a–d)** and diakinesis **(e–g)** chromosomes of *T. cinerariifolium* (2*n* = 18). **(a)** Rabl formation showed with telomeric sequence TTTAGGG (red) and TcSAT1 (green) as probes. **(b–g)** TcSAT1 (green) and/or TcSAT2 (red). **(a,g)** accession Africa-7, **(c,d)** accession 32-2, **(e,f)** accession 53-3. Arrow in (**b–d)** mark association of TcSAT1 and TcSAT2 sites. Bivalents in **(f)** are arranged according to metaphase chromosomes shown on the Figure 2p. Arrow in **(f)** points to a bivalents that show heterozygosity of subtelomeric heterochromatin. Arrow in **(g)** indicates connection between non-homologous chromosomes. Scale bar, 10 μm.

FISH on meiotic chromosomes of *T. cinerariifolium* using TcSAT1 and TcSAT2 satellite DNA repeats and *Arabidopsis*-type (TTTAGGG)_n_ telomeric repeats as probes was performed using accessions 20-7, 32-2, 53-3 and Africa-7 selected for their flower buds availability (Table 1 and Figure 5). In somatic nuclei from anther layers surrounding early meiocytes, Rabl configuration is evident, showing pericentromeric and interstitial telomeric signals at one side of nuclei and telomeric and subtelomeric signals on opposite side of the nuclei (Figure 5a). In pachytene, a large cluster of subtelomeric heterochromatin containing up to nine telomeric signals is observed (Figure 5b–d, arrow). When the telomere red signals are surrounded by large green subtelomeric heterochromatin blocks they are observed as orange-yellow due to color superposition. In diakinesis, mostly rod bivalents were formed. Terminal chiasmata were formed on all chromosome pairs, on both or on one of the chromosome ends (Figure 5e,f). Subtelomeric heterochromatin connections between bivalents were observed in some cells in diakinesis (arrowed in Figure 5g). Subtelomeric heterochromatin connections correspond to associations between TcSAT1 and TcSAT2 sequences from non-homologous chromosome pairs. The frequency of cells showing these connections is difficult to estimate because it was difficult to obtain in fully spread nine bivalents, especially because _“_squash” technique has not been used in this study. However, chromosome connections were evident in more than 50% of cells in diakinesis.

## Discussion

We present the first comprehensive characterization of repetitive DNA composition of *Tanacetum cinerariifolium* genome. We found that retroelements dominate in the genome of *T. cinerariifolium* as in other investigated plant genomes (González et al., 2018). The genome size (1C) determined previously for 17 diploid *Tanacetum* taxa ranges from 1.92 to 6.595 pg (Olanj et al., 2015). Thus, *T. cinerariifolium* (1C = 9.58 pg) has by far the highest genome size of all known *Tanacetum* diploids. In *T. cinerariifolium*, genome expansion can be a result of accumulation of retroelements. However, the hypothesis that genome expansion could partly also be related to ITR amplification should be taken into consideration due to the large ITR blocks present in the genome of *T. cinerariifolium*. Most plant satDNA sequences commonly have monomer unit lengths of about 150–400 bp (Mehrotra and Goyal, 2014; Garrido-Ramos, 2017). In *T. cinerariifolium*, satellite DNA monomeric sizes of 897 bp (TcSAT2) and 1120 bp (TcSAT3) are rather untypical, but not unique. SatDNAs with repeat lengths that are longer than 800 bp are also reported in other species like *Brassica nigra, Hordeum chilense, Rumex, Secale cereale* or *Phaseolus vulgaris* (for a review see Mehrotra and Goyal, 2014).

### Genomic Organization of the 35S and 5S rDNAs

*Tanacetum cinerariifolium* exhibits a linked rDNA configuration (L-type). The linked organization of 35S-5S rDNA units has been reported within three large groups of the subfamily Asteroideae: tribe Anthemideae (93% of the studied cases, i.e., 56 species), tribe Gnaphalieae (100%, i.e., 10 species) and in the “Heliantheae alliance” (23%, i.e., 4 species) (Garcia et al., 2010). In *T. cinerariifolium*, like in most L-type genomes, the 5S rDNA insertion occurs in the IGS region within 1 kb downstream from the 26S gene, and the corresponding transcript is encoded exclusively on the opposite DNA strand than the 26 S rRNA (Garcia et al., 2010; Mazzella et al., 2010). 332 bp long nuclear rDNA intergenic spacer IGS1 of *T. cinerariifolium* fits well within the range of all IGS1 types found so far in L-type arranged plant species, which range from 210 bp in *Tagetes minuta* to 454 bp in *Artemisia absinthium* (Garcia et al., 2010). The hypothesis involving retrotransposition event that gave rise to linked rDNAs arrangement has been previously proposed to explain the appearance of linked rRNA genes in the subfamily Asteroideae (Garcia et al., 2010). This hypothesis is supported by the presence of terminal inverted repeats (TIR) of Cassandra retroelement found in the IGSs of many L-type species. We did not find Cassandra retroelements signatures in the IGS of *T. cinerariifolium*, however, 34 bp long leftover of MITE (non-autonomous DNA transposon remnant) has been found in the IGS of *T. cinerariifolium*.

### Interstitial Telomeric Repeats

FISH revealed the presence of large interstitial telomeric-like repeats (ITRs) in *T. cinerariifolium* suggesting that ITRs represent a significant part of the telomeric DNA in *T. cinerariifolium*. Each homolog chromosome pair possessed unique ITRs pattern which facilitated identification of all chromosome pairs in the complement. ITRs have been reported in other species of this genus, *Tanacetum ptarmiciflorum* (Shibata and Hizume, 2011), as well as in two other genera of Anthemideae (*Matricaria* and *Anacyclus*) that are closely related to *Tanacetum* (Sonboli et al., 2012; Vitales et al., 2018). In contrast, no ITRs were detected in four species of *Argyranthemum* (Borgen et al., 2003), a genus also belonging in the tribe Anthemideae, but distantly related to *Tanacetum* (Oberprieler et al., 2009). In order to explain scattered distribution of ITRs among the Anthemideae, Rosato et al. (2018) proposed a hypothesis that ITRs originated early in the diversification of the Eurasian clade of Anthemideae (Oberprieler et al., 2009) and were followed by complex and recurrent cycles of amplification, genomic spread and contraction.

The occurrence of ITRs outside of the chromosomal termini is not fully understood. A four-step mechanism to explain the presence of this kind of ITRs was proposed for vertebrate chromosomes (Ruiz-Herrera et al., 2008), and is in line with the “centromere-from telomere” hypothesis by Villasante et al. (2007). According to this hypothesis ITRs in centromeric/pericentromeric regions represent remnants of structural chromosome rearrangements that occurred during karyotypic evolution, such as Robertsonian (Rb)-like fusions, tandem chromosome fusions or pericentric inversions. The majority of the species of the Anthemideae tribe have basic chromosome number *x* = 9, while *x* = 8-based species have been found in the *Artemisia* genus suggesting that Robertsonian translocations might have participated in the evolution of some species of this tribe (Garcia et al., 2006; Mas de Xaxars et al., 2016). However, this hypothesis of ITRs origin in the Anthemidae is not the most parsimonious as there was no change in chromosome number neither in *Anacyclus*, nor in *Tanacetum* (both at the ploidy level) (Olanj et al., 2015; Rosato et al., 2018). On the other hand, *Anacyclus* species are characterized by striking variability of ITR sites (Rosato et al., 2018). Furthermore, in *T. cinerariifolium* ITRs are present not only in pericentric regions but also in interstitial positions, while in the sister genus *Anacyclus* ITRs are present exclusively in the interstitial positions (this study and Rosato et al., 2018). Therefore, the results of this and previous study (Rosato et al., 2018) argue that ancient Robertsonian translocations alone cannot explain all of the ITRs patterns in Anthemideae.

Alternatively, ITRs might have originated from the insertion of telomeric repeats during the repair of DSBs during evolution (Ruiz-Herrera et al., 2008). These short ITRs blocks formed in the “chromosome healing” process, could have repeatedly expanded in the fashion that drives microsatellite expansions utilizing DNA polymerase slippage, unequal crossing-over or gene conversion. The comparison of 10 human ITRs loci with their genomic orthologs in 12 primate species showed that short ITRs evolved during primate evolution, not from the expansion of pre-existing telomeric repeats, as is the case for other microsatellites, but rather as new insertions of telomeric repeats in pre-existing and well conserved unrelated sequences (Nergadze et al., 2007).

Several studies have also shown that the presence of ITRs in plant chromosomes might simply reflect the fact that these telomeric sequences are a component of specific tandem DNA repeats (Tek and Jiang, 2004; Mlinarec et al., 2009; Emadzade et al., 2014). In these cases, ITRs are assumed to be the result of amplifications of telomeric repeats that occurred independently during the chromosomal evolution of species. In *T. cinerariifolium*, no co-localization of ITRs with the three satellites repeats, TcSAT1, TcSAT2 and TcSAT3, have been found. There is also a possibility that ITRs are component of some other, yet unidentified satellite repeats which may be not as abundant as the TcSAT1/TcSAT2 families and thus were underrepresented in the current analyses or might be underrepresented due to technical reasons (Weiss-Schneeweiss et al., 2015). However, this hypothesis is less likely because ITRs formed large blocks in the *T. cinerariifolium* genome and if this indeed is the case, then ITRs should be a part of highly abundant satDNA families and therefore should be found in bioinformatic analyses. Further studies are needed to determine the origin and to identify the mechanism underlying the evolutionary dynamics of ITRs in the chromosomes of Anthemidae during their karyotype evolution.

### Dynamics of the DNA Sequences in the Subtelomeric Regions

In this study, we showed that subtelomeric regions of *T. cinerariifolium* are highly polymorphic providing strong evidence for the dynamic nature in the subtelomeric regions. We detected eight polymorphic TcSAT1 loci and 14 polymorphic TcSAT2 loci by analyzing karyograms of only 12 individuals. Subtelomere dynamics is not confined to *T. cinerariifolium*. Several *Tanacetum* species native to Iran showed high polymorphism in the number of GC-rich (CMA+) bands and rDNA loci (Olanj et al., 2015) suggesting that polymorphic nature of subtelomeric regions is probably characteristic of the whole genus.

Polymorphic nature of the subtelomeric regions has been reported in other groups of organisms such as humans, yeast and the malaria parasite *Plasmodium* (Louis, 1995; Freitas-Junior et al., 2000; Linardopoulou et al., 2005). In plants, plasticity of subtelomeres has rarely been reported, only for a few sequenced plant species such as rye, potato and common bean (Vershinin et al., 1995; González-García et al., 2006; Torres et al., 2011; Richard et al., 2013; Chen et al., 2018). For example, subtelomeres from the model plant *Arabidopsis* present simple organization (Heacock et al., 2004; Kuo et al., 2006). However, in contrast to previous findings, this is the first report showing dynamic nature of subtelomeric heterochromatin in plants demonstrated on the basis of detailed analyses of the chromosomal distribution of polymorphic satDNAs sites on a significant number of populations and individuals.

The unequal cross over is probably the most parsimonious explanation for the occurrence of polymorphic TcSAT1 and TcSAT2 sites at the subtelomeres of *T. cinerariifolium*. An abundance of cytological observation indicates that initiation of homologous chromosome synapsis and recombination occurs toward the chromosome ends (Scherthan, 2001; Schwarzacher, 2003). Terminal chiasmata observed in all bivalents of *T. cinerariifolium* suggest that in this species homologous recombination is frequent in subtelomeric regions. The large differences in subtelomeric heterochromatin between homologous chromosomes are obviously not an impediment to form the chiasma. Furthermore, both homologs show similar size and intensity of telomeric signals, in spite of the large subtelomeric heterochromatin block present in only one of the homologous chromosomes.

In addition, the TcSAT1 and TcSAT2 expansion could have been driven by ectopic recombination. The fact that the TcSAT1 and TcSAT2 are found at most *T. cinerariifolium* terminal knobs might indicate frequent exchanges between *T. cinerariifolium* subtelomeric regions. Connections between non-homologous chromosomes observed in *T. cinerariifolium* meiocytes provide further support for this hypothesis. Subtelomeric heterochromatin connections very likely arose from the subtelomeric heterochromatin clumps observed at pachytene. The association of subtelomeric heterochromatin from non-homologous chromosomes is not an optical effect produced either by squashing or chromosome bending, as squashing was not used in this work. In *T. cinerariifolium*, the large differences in subtelomeric heterochromatin exist almost between every (if not all) homologous chromosome pairs. A major body of work reveals that if the alleles at two loci are heterozygous, then ectopic recombination is relatively likely to occur due to the absence of homologous template for its repair, whereas if the alleles are homozygous, they will almost certainly undergo allelic recombination (Linardopoulou et al., 2005). DSBs occurring in a hemizygous region stemming from an unbalanced translocation increases probability of causing another translocation, inversion or intrachromosomal deletion (Linardopoulou et al., 2005). Generation of highly variable regions in subtelomeric heterochromatin by ectopic recombination has been reported in other plant species such as rye, *Aegilops* and in common bean (Rashkina et al., 2004; González-García et al., 2006; Richard et al., 2013; Chen et al., 2018). In *Phaseolus vulgaris* subtelomeres are hot spots of both intra- and inter-chromosomal recombination (Chen et al., 2018).

Additional biological factors may be associated with the generation and maintenance of the polymorphism of the subtelomeric regions in *T. cinerariifolium*. The heterozygosity found in every *T. cinerariifolium* individual is likely to be contributed to by outcrossing given that *T. cinerariifolium* is an obligate outcrosser (MacDonald, 1995). In the Asteraceae, outcrossing is promoted by the sporophytic self-incompatibility (SSI) (Allen and Hiscock, 2008). Therefore, it seems that many evolutionary processes might be shaping the amount and organization of the subtelomeric regions in *T. cinerariifolium*.

With their highly dynamic nature, subtelomeres could serve as a place where haplotypes can diversify faster than in single-copy genomic regions. Sequence transfer between non-homologous chromosomes has the potential to create advantageous new combinations of sequence variants, aiding adaptive peak shifts (Linardopoulou et al., 2005). Subtelomeres of most eukaryotes contain fast-evolving genes usually involved in adaptive processes (Chen et al., 2018). For example, in *Phaseolus vulgaris* subtelomeres favor the rapid evolution of resistance genes (Chen et al., 2018). Subtelomeric regions have been associated with adaptive processes in *Tanacetum* and *Myopordon* genera from the Asteraceae family. *Tanacetum* species living at higher altitudes tend to present more GC-rich DNA (Olanj et al., 2015). In line with this hypothesis, Hidalgo et al. (2008) found a large number of heterochromatic bands (both GC- and AT-rich) in species from the Asteraceae genus *Myopordon* Boiss. inhabiting high mountain areas.

This work is the most comprehensive characterization of repetitive DNA composition of *Tanacetum cinerariifolium* genome. We have confirmed linkage of 5S and 35S rDNAs in all chromosomal loci. We showed large interstitial telomeric-like repeats (ITRs) present in all *T. cinerariifolium* chromosomes. We also demonstrated that, in contrast to stable ITRs pattern, subtelomeres of *T. cinerariifolium* are highly polymorphic. For the first time, to our knowledge, high levels of DNA repeats polymorphism at the subtelomeric regions in diploid plants are demonstrated on the basis of detailed FISH analysis on a significant number of populations and individuals. We propose a mechanism such as unequal crossing-over and non-homologous recombination being implicated in the origin of such polymorphism.

## Data Availability

Publicly available datasets were analyzed in this study. This data can be found here: http://www.ncbi.nlm.nih.gov/bioproject/524009.

## Author Contributions

JMl and VB designed the study. JMl interpreted the results, wrote the manuscript, and carried out the cytogenetic experiments with the help of AS, AJ, and VB. JMc and HW-S carried out the bioinformatic studies. HW-S drafted the manuscript. BB carried out flow cytometry with the help of JMl and NM. All authors read and approved the final manuscript.

## Conflict of Interest Statement

The authors declare that the research was conducted in the absence of any commercial or financial relationships that could be construed as a potential conflict of interest.

## Footnotes

1 http://www.repeatmasker.org

2 http://genedenovoweb.ticp.net:81/DPTEdb/index.php

## Supplementary Material

FIGURE S1 Analysis of three major tandem repeats in the *T. cinerariifolium* genome. Star-like or circular graph layouts of read clusters indicative of *T. cinerariifolium* tandem repeats. The graph layouts corresponding to read clusters of **(a)** TcSAT1, **(b)** TcSAT2, and **(c)** TcSAT3 are represented. The percentage indicates the genome proportion of each cluster.

FIGURE S2 Consensus sequences of TcSAT1, TcSAT2 and TcSAT3 repeats in fasta format. Primer sequences for amplification of tandem repeat specific probes are underlined. The regions used as a probe are marked in bold.

FIGURE S3 Consensus sequences of TcSAT1 **(a)**, TcSAT2 **(b)** and TcSAT3 **(c)** repeats inferred from fragments reconstructed from the most frequent 27-mers (TcSAT1), 23-mers (TcSAT2) or 19-mers (TcSAT3) detected in NextSeq sequence reads. The consensus is displayed as sequence logo where the height of the letters corresponds to frequencies of corresponding k-mers. Major sequence variants are displayed along with the prevailing bases. Detailed information about reconstructed fragments used to create the consensus logos are provided as Table S3.

FIGURE S4 Dot-plot analysis of TcSAT1, TcSAT2, and TcSAT3 repeats in regard to the presence of subrepeats.

FIGURE S5 FISH mapping of TcSAT1 and TcSAT2 satellite DNA repeats on somatic metaphase chromosomes of *Tanacetum cinerariifolium* (2*n* = 18, accession 32-2). **(a)** chromosomes counterstained with DAPI. **(b–d)** FISH mapping of TcSAT1 (green) and TcSAT2 repeats (red). Arrows in **(a)** point DAPI-positive heterochromatin.

## Abbreviations

DSB: double strand break
FISH: fluorescence *in situ* hybridization
IGS: 26S-18S intergenic spacer
ITRs: interstitial telomeric repeats
MITE: miniature inverted-repeat transposable elements
rDNA: ribosomal DNA
TcSAT1: *Tanacetum cinerariifolium* satellite 1
TcSAT2: *T. cinerariifolium* satellite 2
TcSAT3: *T. cinerariifolium* satellite 3
