# Supplementary figures and images for: The Repetitive DNA Composition in the Natural Pesticide Producer Tanacetum cinerariifolium: Interindividual Variation of Subtelomeric Tandem Repeats
Source: Front Plant Sci. 2019 May 16;10:613. doi: 10.3389/fpls.2019.00613 (PMC6532368; doi:10.3389/fpls.2019.00613)

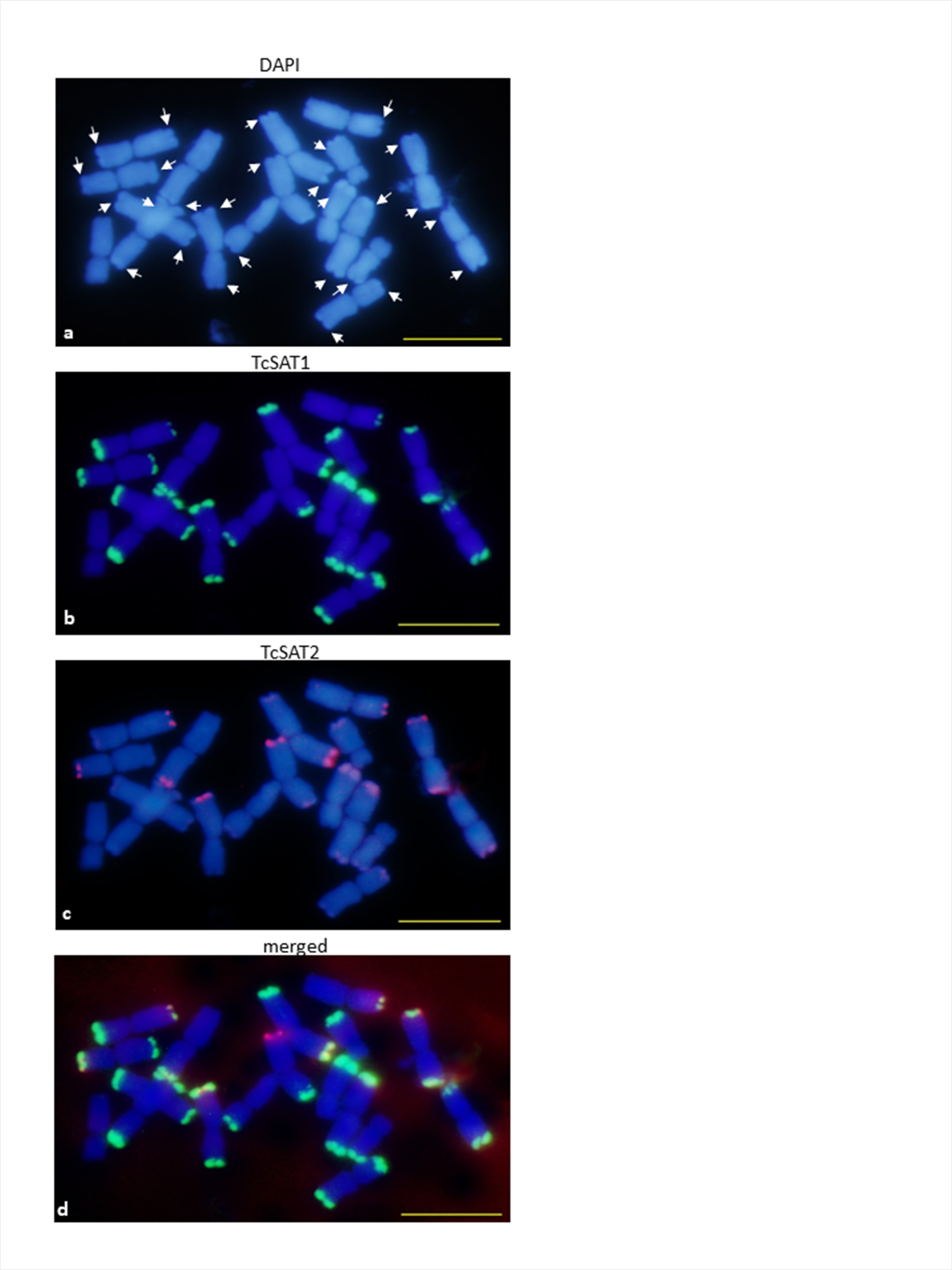

Supplement: FIGURE S1 — Analysis of three major tandem repeats in the T. cinerariifolium genome. Star-like or circular graph layouts of read clusters indicative of T. cinerariifolium tandem repeats. The graph layouts corresponding to read clusters of (a) TcSAT1, (b) TcSAT2, and (c) TcSAT3 are represented. The percentage indicates the genome proportion of each cluster. [file Supplementary_Figure_5.TIF]

TcSAT1  
0.882%

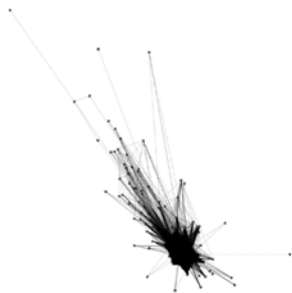

**a**

TcSAT2  
0.116%

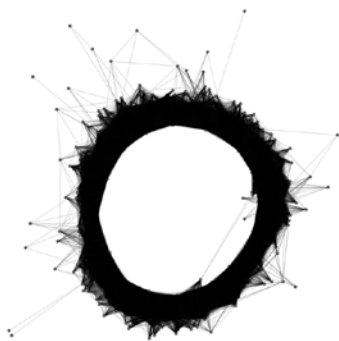

**b**

TcSAT3  
0.016%

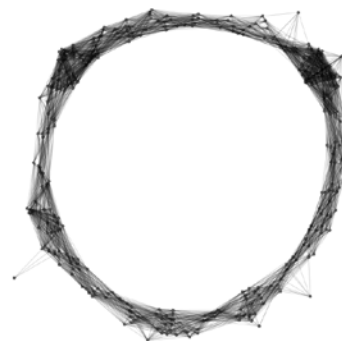

**c**

Supplement: FIGURE S2 — Consensus sequences of TcSAT1, TcSAT2 and TcSAT3 repeats in fasta format. Primer sequences for amplification of tandem repeat specific probes are underlined. The regions used as a probe are marked in bold. [file Supplementary_Figure_1.PDF]

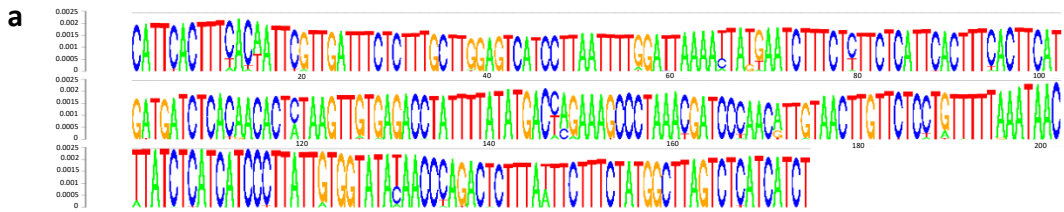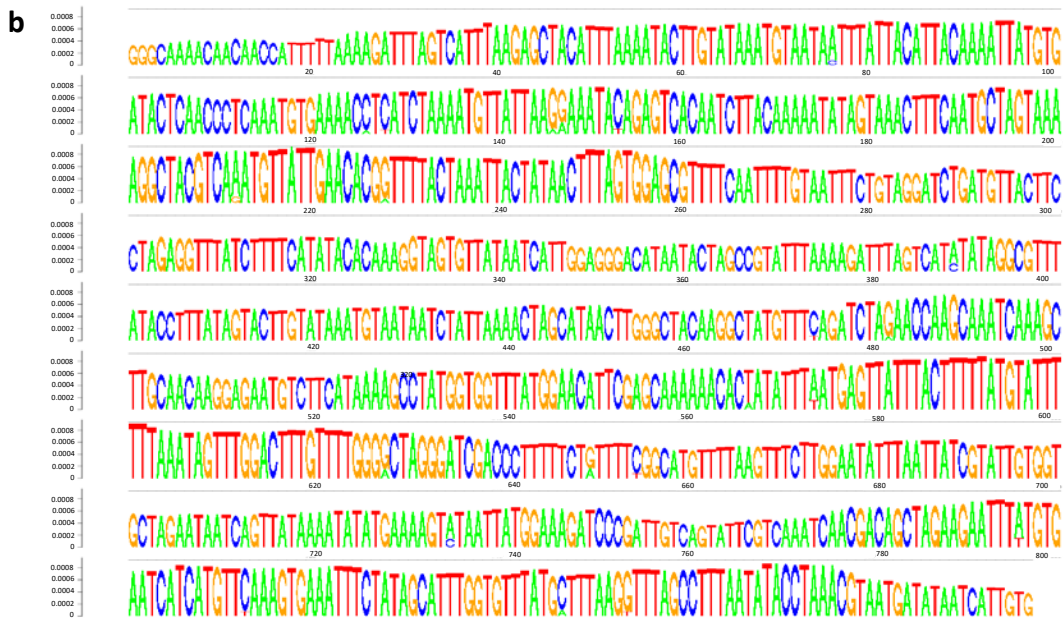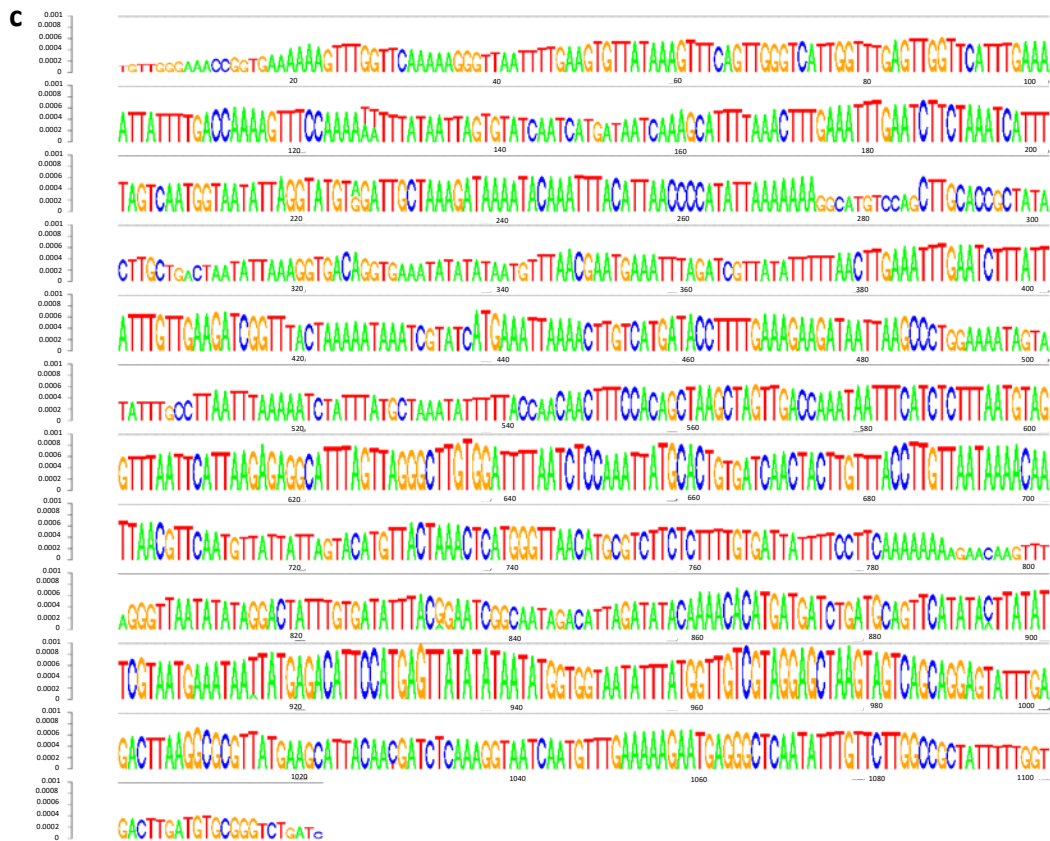

Supplement: FIGURE S4 — Dot-plot analysis of TcSAT1, TcSAT2, and TcSAT3 repeats in regard to the presence of subrepeats. [file Supplementary_Figure_3.PDF]

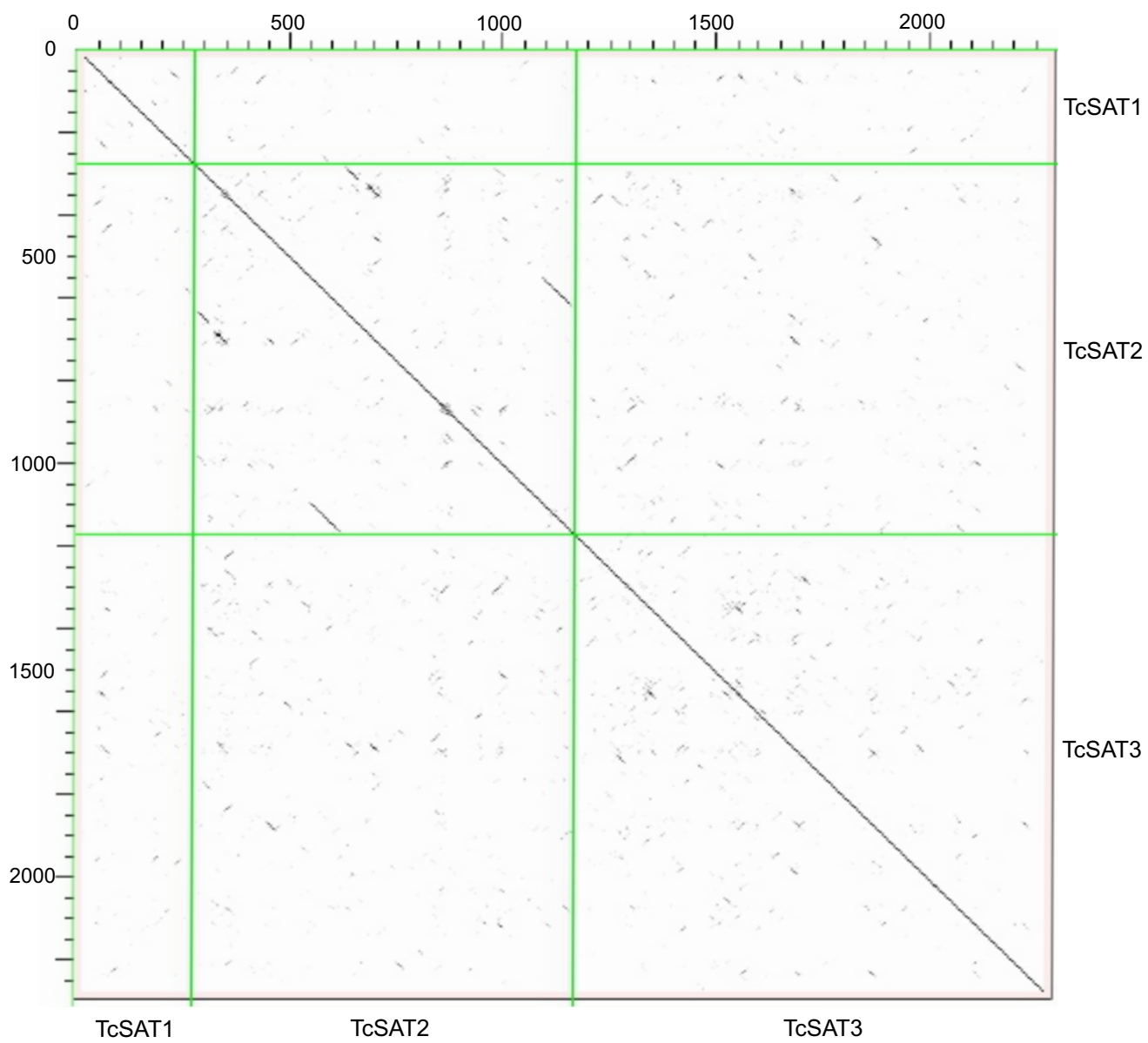

Supplement: FIGURE S5 — FISH mapping of TcSAT1 and TcSAT2 satellite DNA repeats on somatic metaphase chromosomes of Tanacetum cinerariifolium (2n = 18, accession 32-2). (a) chromosomes counterstained with DAPI. (b–d) FISH mapping of TcSAT1 (green) and TcSAT2 repeats (red). Arrows in (a) point DAPI-positive heterochromatin. [file Supplementary_Figure_4.PDF]
