# Supplementary material for: The Repetitive DNA Composition in the Natural Pesticide Producer Tanacetum cinerariifolium: Interindividual Variation of Subtelomeric Tandem Repeats
Source: Front Plant Sci. 2019 May 16;10:613. doi: 10.3389/fpls.2019.00613 (PMC6532368; doi:10.3389/fpls.2019.00613)
Supplement: FIGURE S3 — Consensus sequences of TcSAT1 (a), TcSAT2 (b) and TcSAT3 (c) repeats inferred from fragments reconstructed from the most frequent 27-mers (TcSAT1), 23-mers (TcSAT2) or 19-mers (TcSAT3) detected in NextSeq sequence reads. The consensus is displayed as sequence logo where the height of the letters corresponds to frequencies of corresponding k-mers. Major sequence variants are displayed along with the prevailing bases. Detailed information about reconstructed fragments used to create the consensus logos are provided as Table S3. [file Supplementary_Figure_2.PDF]

>TcSAT1 (CL14)
CATTCACTTTCACAATT**CGTTGATTTCTCTTTGCTTGGAGTCATCCTTAATTTTGGATTAAAATTATGAATCTTTCTCTT**
CTCATTCACTTTCACTTCATGATGATCTCACAACACTCTAAGTTGTGAGACCTATTTTATATGACCAGAAAGCCCTAAAC
GATCCCAACATTGTAACTTGTTCTCCTGTTTTTAAATAACTTATCTCATCATCCCTTATTGTGGTATATAACCCAGACTC
TTTATTTCTTTCTATGGCTTAGTCTCATCATCT

>TcSAT2 (CL82)
GGGCAAAACAACAACCATTTTTAAAAGATTTAGTCATTTAAGAGCTACATTTAAAATACTTGTATAAATGTAATAATTTA
TTACATTACAAAATTATGTGATACTC**AACCCTCAAATGTGAAAACCTCATCTAAAATGTTATTAAGGAAATACAGAGTCA**
CAATCTTACAAAAATATAGTAAACTTTCAATGCTAGTAAAAGGCTACGTCAAATGTTATTGAACACGGTTTTACTAAATT
ACTATAACTTTAGTGGAGCGTTTTCAATTTTGTAATTTCTGTAGGATCTGATGTTACTTCCTAGAGGTTTATCTTTTCAT
ATACACAAAGGTAGTGTTATAATCATTGGAGGGACATAATACTAGCCGTATTTAAAAGATTTAGTCATATATAGGCGTT
ATACCTTTATAGTACTTGTATAAATGTAATAATCTATTAAAACTAGCATAACTTGGGCTACAAGGCTATGTTTCAGATCT
AGAACCAAGCAAATCAAAGCTTGCAACAAGGAGAATGTCTTCATAAAAGCCTATGGTGGTTTATGGAACATTCGAGCA
AAAAACACTATATTTAATGAGTTATTTACTTTTTATGTATTTTTTAAATAGTTTGGACTTTGTTTTGGGGCTAGGGATCGA
CCCTTTTTCTGTTTTCGGCATGTTTTAAGTTTCTTGGAATATTTAATTATCGTATTGTGGTGCTAGAATAATCAGTTATAA
AATATATGAAAAGTATAATTATGGAAAGATCCCGATTGTCAGTATTCGTCAAATCAACGACAGCTAGAAGAATTTATGTG
AATCATCATGTTCAAAGTGAAATTTCTATAGCATTTGGTGTTTATGCTTTAAGGTTTAGCCTTTAATATACCTAAACGTA
ATGATATAATCATTGTG

>TcSAT3 (CL153)
TGTTGGGAAACCGGTGAAAAAAGTTTGGTTCAAAAAGGGTTAATTTTGAAGTGTTATAAAGTTTCAGTT**GGGTCATTGGT**
TTGAGTTGGTTCATTTGAAAATTATTTTGACCAAAAGTTTCCAAAAATTTTATAATTAGTGTATCAATCATGATAATCAA
AGCATTTTAAACTTTGAAATTTGAATCTTCTAAATCATTTTAGTCAATGGTAATATTAGGTATGTAGATTGCTAAAGATA
AAATACAAATTTACATTAACCCCATATTAAAAAAAGGCATGTCCAGCTTGCACCGCTATACTTGCTGACTAATATTAAAG
GTGACAGGTGAAATATATATAATGTTTAACGAATGAAATTTAGATCGTTATATTTTTAACTTGAAATTTGAATCTTTATT
ATTTGTTGAAGATCGGTTTACTAAAAATAAATCGTATCATGAAATTAAAACTTGTCATGATACCTTTTGAAAGAAGATAA
TTAAGCCCTGGAAAATAGTATATTTGCCTTAATTTAAAAATCTATTTATGCTAAATATTTTTACCAACAACTTTCCACAG
CTAAGCTAGTTGACCAAATAATTTCATCTCTTTAATGTAGGTTTAATTCATTAAGAGAGGCATTTAGTTAGGGCTTGTGG
ATTTTAATCTCCAAATTATGCACTGTGATCAACTACTTGTTTACCTTGTTAATAAAACAATTAACGTTCAATGTTATTAT
TAGTACATGTTACTAAACTCATGGGTTAACATGCGTCTTCTCTTTTGTGATTATTTTCCTTCAAAAAAAAGAACAAGTTT
AGGGTTAATATATAGGACTATTTGTGATATTTACGGAATCGGCAATAGACATTAGATATACAAAACACATGATGATCTGA
TGCAGTTCATATACTTATATTCGTAATGAAATAATTATGAGACATTCCATGAGTTATATATAATATGGTGGTAATATTTA
TGGTTGTCGTAGGAGCTAAGTAGTCAGCAGGAGTATTTGAGACTTAAGGCGCGTTATGAAGCATTACAACGATCTCAAAG
GTAATCAATGTTTGAAAAAGAATGAGGGCTCAATATTTGTTCTTGGCCGCTATTTTTGGTGACTTGATGTGCGGGTCTGA
TC
